# An evaluation of physical access barriers to COVID-19 vaccines uptake among persons with physical disabilities in western Kenya

**DOI:** 10.1186/s12889-024-18592-w

**Published:** 2024-04-22

**Authors:** David Omondi Odongo, Esther Osir, Shehu Shagari Awandu

**Affiliations:** 1 Department of Public Health and Community Health & Development, School of Health Sciences, Jaramogi Oginga Odinga University of Science and Technology, Bondo, Kenya; 2 Department of Biomedical Sciences, School of Health Sciences, Jaramogi Oginga Odinga University of Science and Technology, Bondo, Kenya

**Keywords:** COVID-19, Access, Physical disabilities, Physical barriers

## Abstract

**Background:**

Physically disabled persons continue to be discriminated, excluded and neglected based on design of structures and their location. This hampers equitable access to services and disproportionately affect them during a pandemic. This study aimed to evaluate physical access barriers to COVID-19 vaccines among persons with physical disabilities during the COVID-19 pandemic, (March 2020 to March 2022) in Ugenya Sub-county, Siaya County in Western Kenya.

**Methods:**

The study design was cross-sectional. 108 physically disabled participants were selected using systematic sampling technique. Data was collected using structured questionnaires.

**Results:**

Vaccination location (χ^2^ = 95.480, *p* = 0.001), access to the vaccination room (χ^2^ = 84.098, *p* = 0.001) and mobility impaired (χ^2^_=_ 16.168, *p* = 0.001) had statistically significant associations with uptake of COVID-19 vaccine. Income levels, belief in existence of COVID-19, information from mass media and being married increased the odds of becoming vaccinated (AOR = 1.5, 95% CI 0.7–3.4), (AOR = 1.8, 95% CI 0.8-4.0) (AOR = 2.5, 95% CI 1.5–4.2) and (AOR = 2.2, 95% CI 1.3–3.9*)* respectively. The binary logistic regression analysis showed that transport cost and age (*p* = 0.001) had statistically significant associations with COVID-19 vaccine access and uptake. Those who had difficulty in movement and speaking found uptake of COVID-19 vaccine hard (*p* = 0.001).

**Conclusion:**

Marital status, information from reliable sources, belief in existence of COVID-19 were associated with access to and uptake of COVID-19 vaccine. Additionally, nonpayment of transport cost increased the odds of becoming vaccinated. Therefore, mobile health teams should be put in place to reach the physically disabled who are hard-to-leave home. Additionally, reimbursement of amount spent on transportation can be adopted to boost access to healthcare services by the physically disabled persons.

**Supplementary Information:**

The online version contains supplementary material available at 10.1186/s12889-024-18592-w.

## Background

Vaccination is arguably the most impactful public health intervention in offering protection from preventable diseases that claim up to four million lives annually [1]. World Health Organization (WHO) declared COVID-19 caused by SARs-CoV-2 a pandemic and a public health emergency on 11th of March 2020 [2]. Scientists successfully developed several vaccines at an unprecedented pace to combat COVID-19. The efficacy of these vaccines have been shown through reduced adverse outcomes, low intensive unit care hospitalizations, and decreased mortalities rates among the vaccinated persons [3]. The uptake of these vaccines among the physically disabled persons have remained unknown in low and middle income countries making it difficult to track the vaccination rates in this vulnerable populations for equity reasons [1].

Factors such as door entrances of hospital buildings, roads to facilities, sidewalks, corridors, and parking spaces hinder physically disabled persons from accessing and utilizing healthcare services [4]. These factors exacerbate preexisting inequalities between disabled and non-disabled persons during a pandemic. Sadly, these disparities have increased during the COVID-19 pandemic [5]. Consequently, COVID-19 vaccine access has continued to be elusive through the lens of equity and justice, particularly with the already marginalized disabled persons [2].

The literature review revealed that most African countries failed to heed calls by the World Health Organization to integrate the vulnerable cohorts in their COVID-19 vaccination plans [6]. Zimbabwe is among those few countries that included those with disabilities in her COVID-19 vaccination plan [7]. In Nigeria, disabled persons were excluded in the mass testing for those likely to have contracted the virus [5]. Similarly, in Kenya, disabled persons were not given priority in the COVID-19 response plan and aggregated data on the proportion of this cohort that had been vaccinated was also lacking [8]. This suggests discrimination [9], and is against the approach of leaving no one behind advocated for by the Sustainable Development Goals 2030 [6]. This also violated the United Nations Convention on the Rights of Persons with Disabilities (UNCPRD), guide that requires response to COVID-19 to be anchored on the principle of equality to all persons [1].

In Siaya County in Kenya, Ministry of Health gazetted [10] COVID-19 vaccination sites. Ugenya Sub-county had only one facility listed [11]. In this county, proportion of population estimated to have disability was 0.068 [12]. The COVID-19 vaccine administration in the county was to be on first-come-basis. This was projected to result to low uptake of COVID-19 vaccines among physically disabled persons due to poor road networks in Ugenya Sub-county [13] compounded by other known historical barriers these persons have faced in the society [8]. This a setback to enjoyment of the rights of access to healthcare services as enshrined in Article 43a of the Constitution of Kenya [14].

This study aimed to evaluate physical access barriers to COVID-19 vaccines uptake among physically disabled individuals to unravel the knowledge of vaccination coverage gaps. It also aimed to identify the challenges faced by the physically disabled persons during the COVID-19 pandemic. The findings of the study are aimed to offer ground for advocacy by the organizations of persons with disabilities to the relevant authorities to address access-related challenges facing the physically disabled persons.

## Methods

### Study design and setting

**Fig. 1:**
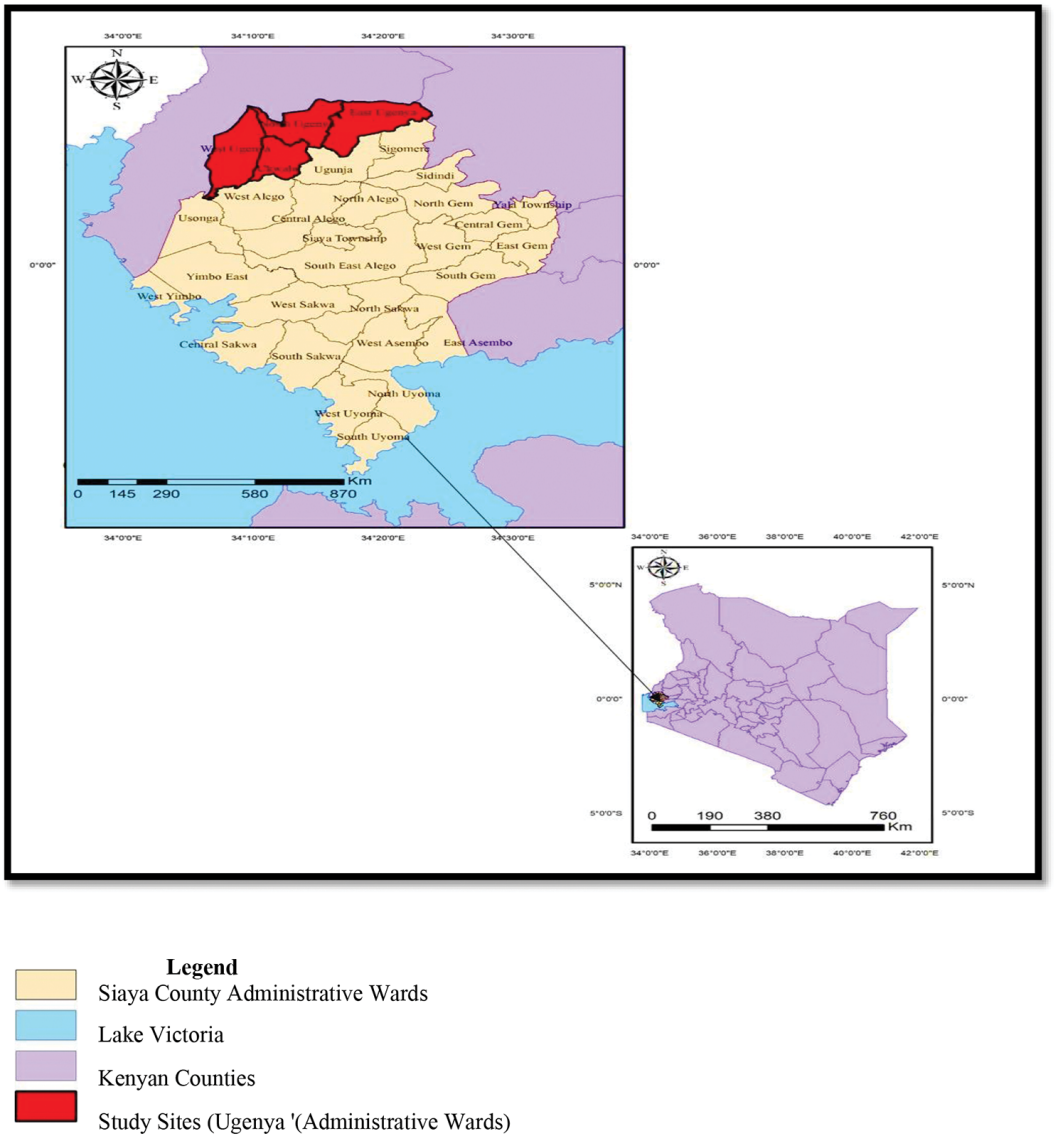
Map of study area

This study used cross-sectional survey design. The setting was Ugenya Sub-county, Siaya County, Western Kenya (Fig. 1). It is one of the six sub-counties in Siaya County. It has four administrative wards namely: West Ugenya, Ukwala, North Ugenya and East Ugenya [9]. It has a land area of 323.5 square kilometer [15]. Each of the wards have the following locations: West Ugenya 9, Ukwala ward 7, North Ugenya ward has 4 and East Ugenya with 5 locations. In this sub-county, total population from 2019 census was 134,354. Out of this population, males were 62,624 while females were 71,726. The proportion of population above 18 was 0.046. Population density in the sub-county was 415 per square kilometers [16]. Ukwala had the highest population density of 350 persons per kilometer square. Participants were recruited in November 2022 and data collected in December 2022.

This map is author-generated using publicly available shape files.

### Participants

The study population were persons with physical disabilities within the community in the four wards of Ugenya Sub-county, Siaya County. The unit of observation and focus was person with physical disability. Only persons with one or more physical disabilities aged 18 years and above were recruited to take part in the study. Forms of disabilities other than physical disabilities were excluded. Those below 18 years were excluded from the study because they were not legible for COVID-19 vaccination at the time of the study. Persons who participated in this study were registered with Kenya National Council of Persons with Disabilities (KNCPWD). Washington Group Short Set (WGSS) were included in the questionnaire to gain more comprehensive view of disability. Respondents used a 4-point Likert-type scale (1 = no difficulty to 4 = cannot do at all) to indicate their difficulty “because of health problem” [1] seeing, even if wearing glasses [3] hearing, even if using a hearing aid [2] walking or climbing stairs.

### Sampling procedure

#### Sample size and sampling procedures

Sample size was determined using Cochran formula [17]. A significance level of 0.05 and confidence level of 95% were used in the study. Proportion used in this study was (0.068) (23), a margin of error (0.05) resulting to 98 individuals. Therefore, the sample size used was 108 after adding a 10% attrition rate. Below is the formula used.

$${\rm{n}}\, = \,{{[{{\rm{Z}}^2}\, \times \,{\rm{P}}(1\, - \,{\rm{P}})]} \over {{{\rm{e}}^2}}}$$

Where n = sample size per group, Z = the critical probability value for 95% confidence level (1.96), P = Proportion used, e = margin of error, (0.05). This formula was used because the outcome variable uptake of COVID-19 vaccine was a categorical variable.

Ugenya Sub-county was stratified into its existing four administrative wards namely: West Ugenya, East Ugenya, Ukwala and North Ugenya. In each of the four administrative wards, 27 participants were targeted. Group leaders of the respective groups of persons with disabilities were purposively identified and approached to help in identifying known persons with physical disabilities. After recruiting the first participant, the other 26 in each ward were selected by systematic sampling. Every 3rd member was recruited until the required sample size was achieved. The selected individuals were approached and their consent requested. Legally authorized representatives of the illiterate participants provided informed consent on behalf of such participants. Participants chose their responses without undue influence from the family members to address internal validity.

### Sample frame used by the study

The population of Ugenya Sub-county was estimated to be 134,354 for the overall population. The population estimated to be above 18 years were 72,872. The eligible persons from the proportion of 0.068 were 4,179. Using the Cochran formula, a sample size of 108 was obtained. This sample size was obtained from 4,179-target population.

### Variables of the study

The outcome variable for this study was uptake of COVID-19 vaccine. Participants were asked if they were vaccinated at least once, and the response category was dichotomized. 1 = Yes and 2 = No. “Yes” responses were considered to have COVID-19 vaccine uptake and “No” responses were considered to have no COVID-19 vaccine uptake.

Socio-demographic and economic data collected were on variables such as age gender, marital status, religion, income, education and employment status. Data collected to evaluate the physical access barriers included distance to the facility, means of transport used to reach the facility, ease of entry into the vaccination room, and willingness to get vaccinated.

### Ethical approval and approval statement

This study was approved by Institutional Review Board of Jaramogi Oginga Odinga University of Science and Technology (JOOUST). Approval number was ERC 33/9/22 − 02. License was obtained from Kenya National Council for Science, Technology and Innovation (NACOSTI), NACOSTI/P/22/21,180. Participants were allowed to read the consent form. Those who could not read on their own had it read to them and interpreted in the local Luo language by the data collectors. Questions and concerns from participants were addressed. They signed the consent form to show their willingness to participate.

### Data collection

The data collectors identified themselves and explained the reason for the visit. Terms of interview were explained. Concerns from the participants were addressed before, during and after the interview. Interviews were conducted in privacy using validated closed-ended questionnaires. Literate participants filled them on their own. Where a participant could not read, write or both, each question was read and interpreted where necessary into local Luo language, and the participant allowed to mark as appropriate without any external interference. Where the participant could not comprehend the questions even after it was translated, a legally authorized representative provided the required information. No identifiers were collected to safeguard privacy of the participants (see Table S1).

### Data analysis and presentations

Data analysis was performed using SPSS version 23 (IBM Corporation). Data on the sociodemographic and economic variables of the study participants were analyzed using descriptive statistics. Frequency distributions and proportions were used to describe and summarize categorical nominal variables. The tests for differences in proportions across categories were performed. The results of the associations were described in terms of odds ratios and significance set at *p* < 0.05 (2-tailed). A binary logistic regression model was used to determine the barriers to COVID-19 vaccine uptake and to control for confounding effects of the independent variables on the outcome.

## Results

### Sociodemographic characteristics of the participants and the vaccination Status

A total of 108 participants took part in this study. The overall COVID-19 vaccines uptake among the participants in this study was 65.7%. However, the number of females vaccinated were more than the number of males vaccinated. Married participants had a higher uptake of COVID-19 vaccines when compared to participants who reported to have been single. Participants who were educated had higher COVID-19 vaccine uptake compared to those who were not educated. The majority of the vaccinated participants received COVID-19 information through mass media while the majority of those not vaccinated received COVID-19 information from family members and relatives. The majority of vaccinated participants believed COVID-19 existed. However, most unvaccinated participants did not believe COVID-19 existed (Table 1). Most vaccinated participants reported narrow doorway as the main challenge they encountered. (see Table S2).

**Table 1:** Sociodemographic and economic characteristics of the participants

Factor	Vaccinated(%)	Not Vaccinated(%)	Total(%)
**Vaccine uptake**	71(65.7%)	37 (34.3%)	108 (100%)
**Gender**			
Male	33(30.6%)	22(20.4%)	55(51.0%)
Female	36(33.3%)	17(15.7%)	53(49.1%)
**Marital status**			
Single	23(46.0%)	27(54.0%)	50(48.0%)
Married	43(79.6%)	11(20.4%)	54(52.0%)
**Education Level**			
None	21(19.4%)	14(13.0%)	35(32.4%)
Primary	37(34.3%)	16(14.8%)	53(49.15%)
Secondary and above	11(10.3%)	8(7.4%)	16(14.8%)
**COVID-19 information**			
Family/Friends	16(44.4%)	20(55.4%)	36(34.6%)
Mass Media	53(79.0%)	15(22.4%)	68(65.4%)
**Disease existence**			
Yes	65 (67.0%)	32(33.0%)	97(89.8%)
No	4(36.4%)	7(63.6%)	11(10.2%)
**Income**			
Earned income	22(31.0%)	16(43.2%)	38(35.2%)
No income	49(69.0%)	21(56.8%)	70(64.2%)

When considering the means of transport to vaccination site, the majority of the participants used motorbike. This was followed distantly by those who used tricycles/wheelchairs or crutches. Very few participants used motor vehicles.

Vaccination site, access to vaccination room and having mobility impairment were strongly associated with uptake of COVID-19 vaccines. The odds of becoming vaccinated was higher among participants who had income compared to those who had no income (AOR = 1.5, 95% CI = 0.7–3.4). Information from mass media increased the likelihood and odds of becoming vaccinated (AOR = 2.5, 95% CI = 1.5–4.2). Belief that COVID-19 existed though had no statistical association with becoming vaccinated, increased odds of becoming vaccinated (AOR = 1.8, 95% CI = 0.8-4.0). Married participants were 2 times more likely to become vaccinated compared to those who were single (AOR = 2.2, 95% CI = 1.3–3.9). The binary logistic regression model showed that interaction between transport cost and age of the participants (χ^2^ = 11.044, *p* < 0.05) was significantly associated with becoming vaccinated. (Table 2).

**Table 2:** Factors associated with COVID-19 vaccine uptake among the participants

Chi-Square Tests				
Factor		Value	Df	P value
Vaccination Site		95.457	2	0.001
Accessing Room		84.098	3	0.001
Mobility Impairment		16.168	3	0.001
**Odds ratios Test**				
**Factor**	**2log likelihood**	**P value**	**Adjusted OR**	**95% CI**
Income	0.9	0.341	1.5	0.7–3.4
Information	11.3	0.002	2.5	1.5–4.2
Disease Existed	3.8	0.05	1.8	0.8–4.1
Marital status	9.4	0.02	2.2	1.3–3.9
**Binary Logistic Regression**				
**Test**		**-2log likelihood**	**Df**	**P value**
Model fitting (Final)		18.164	4	0.001
Goodness of fit		0.0001	65	1.000
Reduced Model (Transport Cost)		12.258	1	0.001

*Note* OR = Odds ratio, CI = Confidence Interval and DF = Degrees of freedom

### Disability types and vaccination status of the participants

When considering the types of disability and vaccination status, those with difficulty in movement and speaking found it hard to access and receive COVID-19 vaccines, *p* = 0.05. However, those with no mobility difficulty and no speech difficulty and those with some difficulty in mobility did not find it difficult getting vaccinated. (Table 3).

**Table 3:** Parameter estimates for the interaction between disability types and vaccination status of the participants

Vaccination Status	B	Wald	Df	P Value
Intercept Mobility1*Speech1 Mobility2*Speech1 Mobility2*Speech4 Mobility3*Speech1 Mobility3*Speech2	-18.367 18.590 19.753 36.681 19.524 0.000	1670.587 530.088 758.471 0.000 1186.006 0.000	1 1 1 1 1 1	0.001 0.001 0.001 0.995 0.001 1.000

*Note* 1 = No difficulty. 2 = Some difficulty. 3 = Much difficulty. 4 = Cannot at all

## Discussion

Here, we explored the physical access barriers to COVID-19 vaccines among persons with physical disabilities during the COVID-19 pandemic. An almost equal proportion of males (33.3%) and females (30.6%) were vaccinated. The Kenya Government through Ministry of Health during COVID-19 vaccination campaigns managed to boost confidence of the public on the safety and efficacy of the vaccines and dispelled misconceptions that were going viral in the communities regarding the safety of the vaccines. The finding is inconsistent with a study in Nigeria where more females were vaccinated than males (25).

Married participants had higher COVID-19 vaccine uptake compared to their unmarried counterparts. It could be because of the involvement of their partners in ensuring that they got vaccinated. In addition, the married participants could have felt the need to keep themselves and their families’ safe from the severity of the COVID-19 in the event of an infection by SARs-CoV-2. Most vaccinated participants reported having received COVID-19 information from mass media. The odds of becoming vaccinated among these participants almost tripled compared to those participants who received the information from friends or relatives (Table 2).

It was because information the information from mass media was clear and credible to them and that the campaigns to boost COVID-19 vaccines uptake were mainly channeled through the mass media. The participants may have been convinced that the COVID-19 vaccines were safe and efficacious in combating COVID-19. The finding is in agreement with the World Health Organization Technical Advisory Report of 2020 that credible information from a trusted source promotes vaccine acceptance and uptake [18]. In addition, family members or friends could have instilled fear to the participants from the rumors regarding the safety of the vaccines.

Participants who believed COVID-19 existed were twice as likely as those who did not believe the disease existed to get vaccinated (Table 2). These persons could have seen people getting vaccinated on the television screens, heard from the radio that COVID-19 vaccines were available and were safe or even witnessed a patient in a critical condition or mortalities from COVID-19. They therefore felt threatened and thus sought vaccination to avert such severities from occurring in their own lives or households in the event of an infection.

**Fig. 2:**
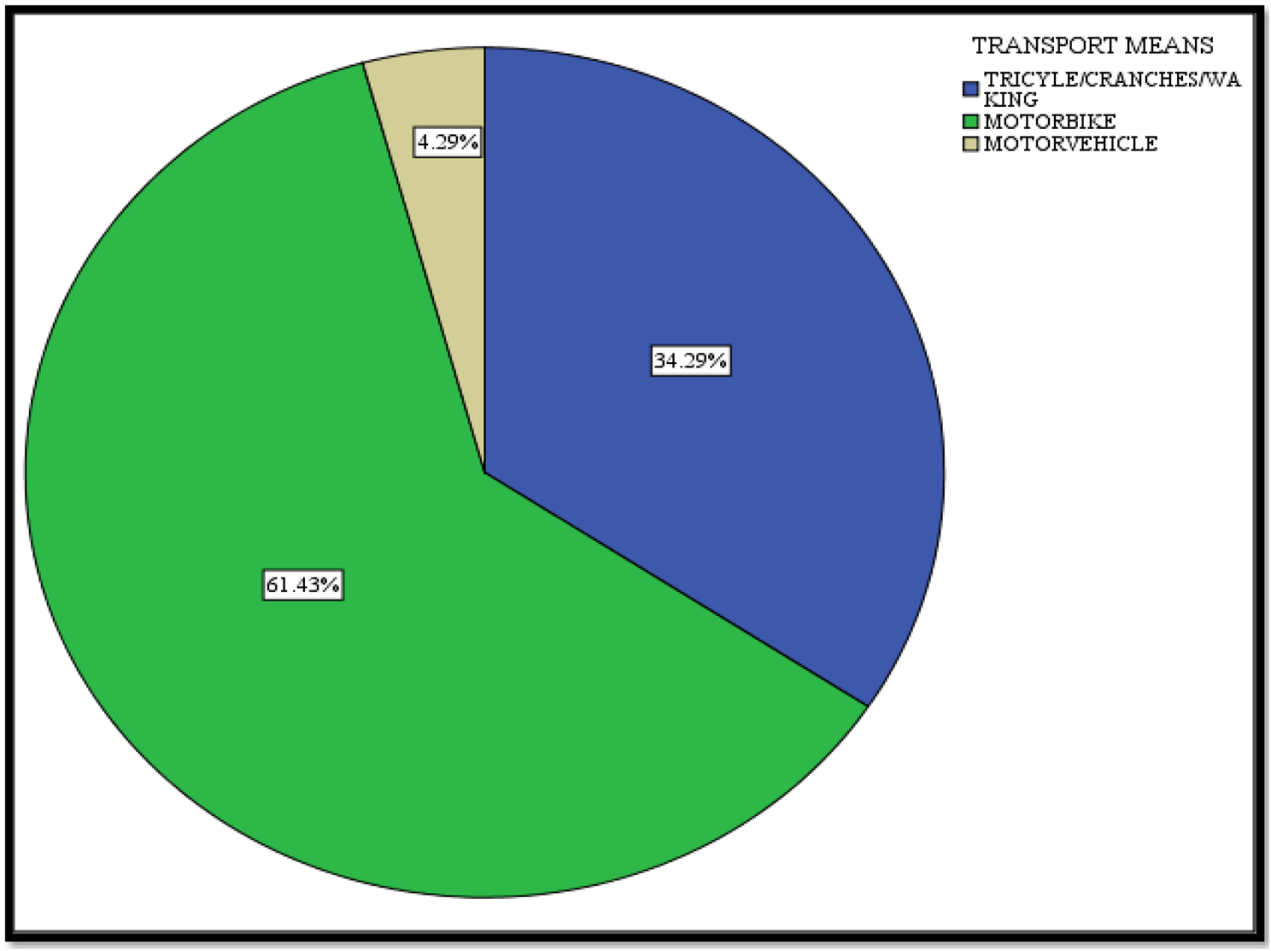
Means of transport to reach vaccination site among physically disabled participants

Additionally, participants who reported to have had income during the COVID-19 pandemic registered a higher COVID-19 vaccine uptake than those with no income. Those who had income could afford transport cost or had acquired walking aids to get to a vaccination facility. It has been further highlighted in Fig. 2 that the highest mode of transport used by the participants to get to the vaccination facility were the motorcycles which had to be paid for. This is consistent with the finding by Harrison and colleagues in Malawi that lack of transport has a strong correlation to limiting access to health facilities among the physically disabled persons [4]. The World Health Organization similarly revealed that transportation is inaccessible to the physically disabled in low and middle- income countries [1].

Vaccination site was associated with becoming vaccinated among the participants (Table 2). Participants were more likely to register higher uptake of COVID-19 vaccines from the nearby health facilities when compared to the distant ones. It shows that if the number of vaccination places were not increased by the Ministry of Health in Ugenya Sub-county, the COVID-19 vaccine access and uptake would be far too low. In fact, Muchuri and colleagues showed that the possible way of increasing the COVID-19 vaccines coverage were increasing number and availability of vaccination sites [8]. The home vaccination approach used in India and in South Africa showed a strong correlation with increased uptake of COVID-19 vaccines among the physically disabled persons [16]. Therefore, equitable uptake of the healthcare service among the physically disabled as provided for in the Universal Health Coverage policy, the Vision 2030 and Article 43a of the Constitution of Kenya can be guaranteed if services are brought closer to the physically disabled persons [10].

From the binary logistic regression result, the cost of transport was the most significant barrier to the uptake of the COVID-19 vaccines (Table 2). Most vaccinated participants flagged access to the vaccination site as the main impediments because of the transportation logistics. In fact, those who never paid for transportation to reach the vaccination site in this study were eight times more likely to be vaccinated compared to the participants who paid for transportation to reach the vaccination site. This suggests that vaccinated rates would be higher among the study participants if mobile teams were available to vaccinate the physically disabled persons in their area of residence as shown by Hashemi and colleagues in Ethiopia [9]. The participants who had mobility and multiple physical impairments were the most disproportionately impacted by transport cost in this study. The lack of wheelchairs, eyeglasses, sunscreen and hearing aids could explain why multiple impaired participants had low uptake of COVID-19 vaccines. The finding was in agreement with the highlight from a study by Orangi and colleagues where lack of mobility and hearing aids reduced health care access among those with multiple disabilities [2].

Majority of the vaccinated participants reported encountering barriers at the facility. Most participants cited narrow entrance and delayed support as the main barriers encountered. (Supplementary file 1). A study by Owuocha and colleagues similarly showed that most participants reported having met the aforementioned challenges in accessing and utilizing healthcare services in western Kenya [10]. This study similarly identified major hindrances of access to services among the physically disabled as the step staircases, steep ramps, and narrow doors and is consistent with the findings of Epstein and colleagues [19].

This study provides baseline information for further research. It also adds to the body of literature on the COVID-19 vaccine access situation for the physically disabled. It informs on the coverage inequalities and the key barriers that still need attention of the stakeholders. The face-to-face interviews conducted helped to increase credibility of the data collected since there was translations into local languages. This made the process more flexible. The study has further revealed that participants with multiple disabilities had the lowest access and uptake to COVID-19 vaccines. This could provide a baseline for further research to establish the extent of their inaccessibility.

This study could have suffered from recall bias from the participants. This could lead to omission of some statements or even distortion of information. Also, the sample size used in this study was insufficient as it was calculated from the proportion of the disabled persons in Siaya County but not from estimate of the barriers to COVID-19 vaccine uptake among the physically disabled persons as it was missing at the time of study. This may have made the study insufficiently powered to assess the physical barriers to COVID-19 vaccines uptake among the physically disabled persons who took up the vaccines and those who did not. However, the results of this study are generalizable because the participants were selected according to a random starting point in the sub-county.

## Conclusion

This study shows that transport cost, narrow entry to the facility and delayed support were significant barriers to COVID-19 vaccine uptake among participants. Mass media appeals to most listeners therefore, the barriers due to misinformation can be countered through mass media. Individuals with multiple physical disabilities still face insurmountable access challenges to centered healthcare services. These challenges hamper equitable access to health care services for the physically disabled persons compared to the general population. These challenges pose threats to the approach of leaving no one behind and attaining universal health coverage. Therefore, mobile healthcare teams should be put in place to reach and to provide services persons with physical disabilities.

### Electronic supplementary material

Below is the link to the electronic supplementary material.

Supplementary Material 1: Raw data of participants

Supplementary Material 2: Sociodemographic and economic characteristic of participants

## Data Availability

The data are available in supplementary files.

## Abbreviations

COVID-19: Corona Virus Disease-2019
ICU: Intensive Care Unit
MoH: Ministry of Health
PWDs: Persons with Disabilities
SDG: Sustainable Development Goal
WHO: World Health Organization
