# Supplementary material for: An evaluation of physical access barriers to COVID-19 vaccines uptake among persons with physical disabilities in western Kenya
Source: BMC Public Health. 2024 Apr 22;24:1112. doi: 10.1186/s12889-024-18592-w (PMC11036731; doi:10.1186/s12889-024-18592-w)
Supplement: Supplementary file 1 — Supplementary Material 1: Raw data of participants [file 12889_2024_18592_MOESM1_ESM.pdf]

SUPPLEMENTARY TABLE 2: SOCIO-DEMOGRAPHIC AND ECONOMIC CHARACTERISTICS OF PARTICIPANTS.

Factor	Vaccinated (%)	Not Vaccinated (%)	Total (%)
**Employment status**			
Not Employment	59(54.6%)	33(30.6%)	92(85.2%)
Employment	10(9.3%)	15(13.9%)	16(14.8%)
**Mobility impaired**			
No	7(6.5%)	8(7.4%)	15(13.9%)
Mild	19(17.6%)	4(3.7%)	23(21.3%)
Moderate	35(32.4%)	12(11.1%)	47(43.5%)
Severe	8(7.4%)	15(13.9%)	23(21.3%)
**Hearing impaired**			
No	53(49.1%)	27(25.0%)	80(74.1%)
Mild	11(10.2%)	5(4.6%)	16(14.8%)
Moderate	4(3.7%)	6(5.6%)	10(9.3%)
Severe	1(0.9%)	1(0.9%)	2(1.9%)

**Visually impaired**			
No	45(41.7%)	33(30.6%)	78(72.2%)
Mild	16(14.8%)	3(2.8%)	19(17.6%)
Moderate	5(4.6%)	2(1.9%)	7(6.5%)
Severe	3(2.8%)	1(0.9%)	4(3.7%)
**Speech difficulty**			
No	64(59.3%)	32(29.6%)	96(88.9%)
Mild	1(0.9%)	2(1.9%)	3(2.3%)
Moderate	1(0.9%)	0(0.0%)	1(0.9%)
Severe	3(2.8%)	5(4.6%)	8(7.4%)
**Decision time**			
1 month	24(34.8%)	1(2.6%)	25 (23.1%)
<3 Months	19(27.5%)	1(2.6%)	20(18.5%)
3-6 Months	9(13.0%)	0(0%)	9(8.3%)
> 6 Months	16(23.2%)	0(0.0%)	16(14.8%)
**Access to room**			
No Difficulty	4(5.8%)	37(94.9%)	41(38.0%)
Narrow Entry	40(58.0%)	2(5.1%)	42(38.9%)
Delayed Support	24(34.8%)	0(0%)	24(22.2%)

Communication Barrier	1(1.4%)	0(0%)	1(0.9%)
**Reception**			
Worst	1(1.5%)	0(0%)	1(1.4%)
Fair	10(14.7%)	1(50.0%)	11(15.7%)
Good	48(70.6%)	1(50.0%)	49(70.0%)
Very Good	9(13.2%)	0(0%)	9(12.9%)

NOTE: < = Less than > = More than
